# Supplementary material for: Heparin-based hydrogel scaffolding alters the transcriptomic profile and increases the chemoresistance of MDA-MB-231 triple-negative breast cancer cells
Source: Biomater Sci. 2020 Feb 13;8(10):2786–96. doi: 10.1039/c9bm01481k (PMC7497406; doi:10.1039/c9bm01481k)
Supplement: Supplementary file 2 [file BM-008-C9BM01481K-s002.zip › Supplementary File 4/HP-B hydrogel + EGF (s)vControl/Pathways/my_analysis.Gsea.1545201170987/HALLMARK_MITOTIC_SPINDLE.html]

Details for gene set HALLMARK\_MITOTIC\_SPINDLE[GSEA]

|  || Dataset | expr.class.cls#HYDEGF\_versus\_CONTROL.class.cls#HYDEGF\_versus\_CONTROL\_repos |
| Phenotype | class.cls#HYDEGF\_versus\_CONTROL\_repos |
| Upregulated in class | HYDEGF |
| GeneSet | HALLMARK\_MITOTIC\_SPINDLE |
| Enrichment Score (ES) | 0.29076058 |
| Normalized Enrichment Score (NES) | 1.3335143 |
| Nominal p-value | 0.025396826 |
| FDR q-value | 0.14654794 |
| FWER p-Value | 0.761 |
Table: GSEA Results Summary

  

Fig 1: Enrichment plot: HALLMARK\_MITOTIC\_SPINDLE      
 Profile of the Running ES Score & Positions of GeneSet Members on the Rank Ordered List

  

| PROBE | DESCRIPTION (from dataset) | GENE SYMBOL | GENE\_TITLE | RANK IN GENE LIST | RANK METRIC SCORE | RUNNING ES | CORE ENRICHMENT || 1 | PIF1 | na |  |  | 364 | 2.291 | -0.0036 | Yes |
| 2 | ESPL1 | na |  |  | 387 | 2.248 | 0.0106 | Yes |
| 3 | CNTRL | na |  |  | 405 | 2.230 | 0.0249 | Yes |
| 4 | KLC1 | na |  |  | 406 | 2.229 | 0.0401 | Yes |
| 5 | DOCK2 | na |  |  | 500 | 2.120 | 0.0496 | Yes |
| 6 | CENPJ | na |  |  | 565 | 2.046 | 0.0602 | Yes |
| 7 | CEP192 | na |  |  | 868 | 1.772 | 0.0563 | Yes |
| 8 | KNTC1 | na |  |  | 938 | 1.725 | 0.0644 | Yes |
| 9 | ALMS1 | na |  |  | 951 | 1.717 | 0.0755 | Yes |
| 10 | CDK5RAP2 | na |  |  | 953 | 1.716 | 0.0871 | Yes |
| 11 | PXN | na |  |  | 974 | 1.702 | 0.0977 | Yes |
| 12 | SPTAN1 | na |  |  | 1042 | 1.658 | 0.1054 | Yes |
| 13 | ALS2 | na |  |  | 1301 | 1.515 | 0.1022 | Yes |
| 14 | CNTROB | na |  |  | 1339 | 1.499 | 0.1104 | Yes |
| 15 | ARHGAP10 | na |  |  | 1388 | 1.472 | 0.1179 | Yes |
| 16 | ATG4B | na |  |  | 1450 | 1.449 | 0.1246 | Yes |
| 17 | INCENP | na |  |  | 1487 | 1.437 | 0.1325 | Yes |
| 18 | RASAL2 | na |  |  | 1606 | 1.389 | 0.1357 | Yes |
| 19 | KIF22 | na |  |  | 1674 | 1.366 | 0.1415 | Yes |
| 20 | CYTH2 | na |  |  | 1688 | 1.362 | 0.1501 | Yes |
| 21 | CTTN | na |  |  | 1713 | 1.355 | 0.1580 | Yes |
| 22 | TUBGCP3 | na |  |  | 1741 | 1.344 | 0.1658 | Yes |
| 23 | PCNT | na |  |  | 1754 | 1.341 | 0.1743 | Yes |
| 24 | TBCD | na |  |  | 1825 | 1.318 | 0.1796 | Yes |
| 25 | CEP250 | na |  |  | 1884 | 1.299 | 0.1854 | Yes |
| 26 | SYNPO | na |  |  | 1915 | 1.287 | 0.1925 | Yes |
| 27 | MYH10 | na |  |  | 2056 | 1.250 | 0.1937 | Yes |
| 28 | PKD2 | na |  |  | 2102 | 1.239 | 0.1998 | Yes |
| 29 | ITSN1 | na |  |  | 2244 | 1.198 | 0.2005 | Yes |
| 30 | KIF15 | na |  |  | 2417 | 1.147 | 0.1992 | Yes |
| 31 | CEP72 | na |  |  | 2482 | 1.132 | 0.2036 | Yes |
| 32 | SMC1A | na |  |  | 2558 | 1.113 | 0.2072 | Yes |
| 33 | SAC3D1 | na |  |  | 2564 | 1.111 | 0.2145 | Yes |
| 34 | CLIP1 | na |  |  | 2578 | 1.109 | 0.2214 | Yes |
| 35 | FARP1 | na |  |  | 2623 | 1.098 | 0.2265 | Yes |
| 36 | ARHGEF11 | na |  |  | 2664 | 1.088 | 0.2318 | Yes |
| 37 | ABL1 | na |  |  | 2791 | 1.054 | 0.2324 | Yes |
| 38 | TUBGCP5 | na |  |  | 2998 | 1.004 | 0.2284 | Yes |
| 39 | MYO9B | na |  |  | 3185 | 0.963 | 0.2252 | Yes |
| 40 | TUBGCP6 | na |  |  | 3270 | 0.948 | 0.2272 | Yes |
| 41 | BCAR1 | na |  |  | 3435 | 0.916 | 0.2248 | Yes |
| 42 | ARAP3 | na |  |  | 3440 | 0.915 | 0.2308 | Yes |
| 43 | CENPF | na |  |  | 3556 | 0.892 | 0.2309 | Yes |
| 44 | BCR | na |  |  | 3608 | 0.883 | 0.2342 | Yes |
| 45 | TAOK2 | na |  |  | 3626 | 0.880 | 0.2393 | Yes |
| 46 | RHOT2 | na |  |  | 3705 | 0.863 | 0.2411 | Yes |
| 47 | KATNB1 | na |  |  | 3719 | 0.861 | 0.2462 | Yes |
| 48 | SHROOM2 | na |  |  | 3748 | 0.856 | 0.2506 | Yes |
| 49 | TPX2 | na |  |  | 3754 | 0.855 | 0.2561 | Yes |
| 50 | MAP3K11 | na |  |  | 3818 | 0.842 | 0.2586 | Yes |
| 51 | ROCK1 | na |  |  | 3850 | 0.838 | 0.2626 | Yes |
| 52 | CLASP1 | na |  |  | 4028 | 0.807 | 0.2588 | Yes |
| 53 | NIN | na |  |  | 4033 | 0.806 | 0.2641 | Yes |
| 54 | BIN1 | na |  |  | 4034 | 0.806 | 0.2696 | Yes |
| 55 | HDAC6 | na |  |  | 4075 | 0.800 | 0.2729 | Yes |
| 56 | CLIP2 | na |  |  | 4110 | 0.795 | 0.2766 | Yes |
| 57 | CSNK1D | na |  |  | 4167 | 0.788 | 0.2790 | Yes |
| 58 | NUMA1 | na |  |  | 4302 | 0.766 | 0.2771 | Yes |
| 59 | BUB1 | na |  |  | 4319 | 0.763 | 0.2815 | Yes |
| 60 | RASA1 | na |  |  | 4413 | 0.747 | 0.2817 | Yes |
| 61 | SMC4 | na |  |  | 4414 | 0.747 | 0.2868 | Yes |
| 62 | RANBP9 | na |  |  | 4518 | 0.732 | 0.2863 | Yes |
| 63 | SEPT9 | na |  |  | 4869 | 0.674 | 0.2725 | Yes |
| 64 | DLG1 | na |  |  | 4883 | 0.670 | 0.2764 | Yes |
| 65 | RAPGEF6 | na |  |  | 4984 | 0.654 | 0.2756 | Yes |
| 66 | NF1 | na |  |  | 5105 | 0.636 | 0.2736 | Yes |
| 67 | TSC1 | na |  |  | 5185 | 0.623 | 0.2737 | Yes |
| 68 | KPTN | na |  |  | 5218 | 0.619 | 0.2762 | Yes |
| 69 | CDC42BPA | na |  |  | 5261 | 0.611 | 0.2782 | Yes |
| 70 | ARHGAP27 | na |  |  | 5296 | 0.606 | 0.2805 | Yes |
| 71 | ARHGAP4 | na |  |  | 5326 | 0.600 | 0.2831 | Yes |
| 72 | EPB41L2 | na |  |  | 5330 | 0.599 | 0.2870 | Yes |
| 73 | LLGL1 | na |  |  | 5339 | 0.598 | 0.2906 | Yes |
| 74 | ARHGEF12 | na |  |  | 5445 | 0.584 | 0.2891 | Yes |
| 75 | PRC1 | na |  |  | 5609 | 0.561 | 0.2843 | Yes |
| 76 | SUN2 | na |  |  | 5625 | 0.558 | 0.2873 | Yes |
| 77 | KIF23 | na |  |  | 5633 | 0.557 | 0.2908 | Yes |
| 78 | KIF2C | na |  |  | 5742 | 0.538 | 0.2887 | No |
| 79 | CKAP5 | na |  |  | 5943 | 0.511 | 0.2817 | No |
| 80 | GEMIN4 | na |  |  | 6060 | 0.495 | 0.2790 | No |
| 81 | SHROOM1 | na |  |  | 6166 | 0.480 | 0.2767 | No |
| 82 | OPHN1 | na |  |  | 6170 | 0.480 | 0.2798 | No |
| 83 | VCL | na |  |  | 6326 | 0.460 | 0.2748 | No |
| 84 | APC | na |  |  | 6402 | 0.450 | 0.2739 | No |
| 85 | BRCA2 | na |  |  | 6406 | 0.449 | 0.2768 | No |
| 86 | TUBGCP2 | na |  |  | 6437 | 0.444 | 0.2783 | No |
| 87 | ANLN | na |  |  | 6438 | 0.444 | 0.2813 | No |
| 88 | ARFGEF1 | na |  |  | 6495 | 0.437 | 0.2813 | No |
| 89 | ARHGDIA | na |  |  | 6680 | 0.413 | 0.2744 | No |
| 90 | AKAP13 | na |  |  | 6971 | 0.376 | 0.2617 | No |
| 91 | NOTCH2 | na |  |  | 7202 | 0.343 | 0.2520 | No |
| 92 | PLEKHG2 | na |  |  | 7289 | 0.331 | 0.2497 | No |
| 93 | FGD4 | na |  |  | 7335 | 0.324 | 0.2495 | No |
| 94 | TUBA4A | na |  |  | 7430 | 0.313 | 0.2467 | No |
| 95 | PLK1 | na |  |  | 7470 | 0.309 | 0.2468 | No |
| 96 | KIF4A | na |  |  | 7539 | 0.300 | 0.2452 | No |
| 97 | CD2AP | na |  |  | 7599 | 0.292 | 0.2441 | No |
| 98 | CENPE | na |  |  | 7608 | 0.290 | 0.2457 | No |
| 99 | ARHGEF7 | na |  |  | 7719 | 0.276 | 0.2418 | No |
| 100 | MAP1S | na |  |  | 7732 | 0.274 | 0.2430 | No |
| 101 | EZR | na |  |  | 7800 | 0.263 | 0.2413 | No |
| 102 | CDC42EP1 | na |  |  | 8205 | 0.222 | 0.2215 | No |
| 103 | SSH2 | na |  |  | 8335 | 0.204 | 0.2161 | No |
| 104 | RACGAP1 | na |  |  | 8576 | 0.177 | 0.2047 | No |
| 105 | MARK4 | na |  |  | 8589 | 0.175 | 0.2053 | No |
| 106 | NUSAP1 | na |  |  | 8930 | 0.130 | 0.1883 | No |
| 107 | PAFAH1B1 | na |  |  | 8956 | 0.125 | 0.1878 | No |
| 108 | NDC80 | na |  |  | 9060 | 0.121 | 0.1832 | No |
| 109 | BIRC5 | na |  |  | 9147 | 0.110 | 0.1795 | No |
| 110 | LMNB1 | na |  |  | 9148 | 0.110 | 0.1802 | No |
| 111 | KIF20B | na |  |  | 9759 | 0.042 | 0.1484 | No |
| 112 | ARHGAP29 | na |  |  | 10024 | 0.017 | 0.1346 | No |
| 113 | ARL8A | na |  |  | 10037 | 0.014 | 0.1341 | No |
| 114 | SASS6 | na |  |  | 10071 | 0.009 | 0.1324 | No |
| 115 | TLK1 | na |  |  | 10081 | 0.008 | 0.1320 | No |
| 116 | CCDC88A | na |  |  | 10112 | 0.003 | 0.1305 | No |
| 117 | MID1 | na |  |  | 10139 | -0.001 | 0.1291 | No |
| 118 | RAPGEF5 | na |  |  | 10175 | -0.005 | 0.1273 | No |
| 119 | ARHGEF2 | na |  |  | 10381 | -0.033 | 0.1167 | No |
| 120 | PCM1 | na |  |  | 10632 | -0.057 | 0.1040 | No |
| 121 | KIF5B | na |  |  | 11200 | -0.123 | 0.0750 | No |
| 122 | CCNB2 | na |  |  | 11220 | -0.126 | 0.0748 | No |
| 123 | KIF1B | na |  |  | 11259 | -0.132 | 0.0737 | No |
| 124 | KIF11 | na |  |  | 11269 | -0.132 | 0.0742 | No |
| 125 | AURKA | na |  |  | 11282 | -0.134 | 0.0744 | No |
| 126 | ABR | na |  |  | 11526 | -0.164 | 0.0628 | No |
| 127 | MYO1E | na |  |  | 11738 | -0.194 | 0.0530 | No |
| 128 | TUBD1 | na |  |  | 12032 | -0.222 | 0.0391 | No |
| 129 | LRPPRC | na |  |  | 12106 | -0.234 | 0.0368 | No |
| 130 | KIF3B | na |  |  | 12188 | -0.242 | 0.0342 | No |
| 131 | EPB41 | na |  |  | 12209 | -0.245 | 0.0348 | No |
| 132 | NCK2 | na |  |  | 12703 | -0.314 | 0.0110 | No |
| 133 | TOP2A | na |  |  | 12732 | -0.319 | 0.0117 | No |
| 134 | HOOK3 | na |  |  | 12977 | -0.336 | 0.0012 | No |
| 135 | DLGAP5 | na |  |  | 13044 | -0.348 | 0.0001 | No |
| 136 | TTK | na |  |  | 13159 | -0.362 | -0.0034 | No |
| 137 | PCGF5 | na |  |  | 13286 | -0.383 | -0.0075 | No |
| 138 | ECT2 | na |  |  | 13375 | -0.398 | -0.0094 | No |
| 139 | NEK2 | na |  |  | 13399 | -0.403 | -0.0078 | No |
| 140 | WASL | na |  |  | 13566 | -0.423 | -0.0137 | No |
| 141 | RAB3GAP1 | na |  |  | 13592 | -0.423 | -0.0121 | No |
| 142 | FBXO5 | na |  |  | 13612 | -0.427 | -0.0102 | No |
| 143 | PPP4R2 | na |  |  | 14007 | -0.490 | -0.0276 | No |
| 144 | WASF2 | na |  |  | 14017 | -0.491 | -0.0247 | No |
| 145 | CEP57 | na |  |  | 14212 | -0.506 | -0.0315 | No |
| 146 | RALBP1 | na |  |  | 14437 | -0.547 | -0.0396 | No |
| 147 | RASA2 | na |  |  | 14724 | -0.593 | -0.0506 | No |
| 148 | DOCK4 | na |  |  | 14796 | -0.602 | -0.0502 | No |
| 149 | WASF1 | na |  |  | 14830 | -0.609 | -0.0478 | No |
| 150 | FSCN1 | na |  |  | 14874 | -0.615 | -0.0459 | No |
| 151 | RABGAP1 | na |  |  | 14883 | -0.616 | -0.0421 | No |
| 152 | MAPRE1 | na |  |  | 14984 | -0.630 | -0.0431 | No |
| 153 | KIF3C | na |  |  | 15097 | -0.651 | -0.0445 | No |
| 154 | CDC42EP2 | na |  |  | 15122 | -0.657 | -0.0413 | No |
| 155 | ARHGEF3 | na |  |  | 15180 | -0.665 | -0.0398 | No |
| 156 | RHOF | na |  |  | 15197 | -0.669 | -0.0361 | No |
| 157 | RFC1 | na |  |  | 15216 | -0.672 | -0.0325 | No |
| 158 | CDC42EP4 | na |  |  | 15230 | -0.674 | -0.0285 | No |
| 159 | MARCKS | na |  |  | 15423 | -0.704 | -0.0339 | No |
| 160 | ABI1 | na |  |  | 15459 | -0.714 | -0.0308 | No |
| 161 | RICTOR | na |  |  | 15461 | -0.714 | -0.0260 | No |
| 162 | SMC3 | na |  |  | 15589 | -0.740 | -0.0277 | No |
| 163 | LATS1 | na |  |  | 15688 | -0.759 | -0.0277 | No |
| 164 | PDLIM5 | na |  |  | 15884 | -0.799 | -0.0325 | No |
| 165 | CDK1 | na |  |  | 15956 | -0.811 | -0.0307 | No |
| 166 | CDC42 | na |  |  | 16013 | -0.823 | -0.0280 | No |
| 167 | ARFIP2 | na |  |  | 16132 | -0.853 | -0.0284 | No |
| 168 | TIAM1 | na |  |  | 16222 | -0.879 | -0.0271 | No |
| 169 | GSN | na |  |  | 16299 | -0.897 | -0.0250 | No |
| 170 | DYNLL2 | na |  |  | 16359 | -0.913 | -0.0219 | No |
| 171 | BCL2L11 | na |  |  | 16361 | -0.913 | -0.0157 | No |
| 172 | PALLD | na |  |  | 16611 | -0.974 | -0.0222 | No |
| 173 | SORBS2 | na |  |  | 16619 | -0.976 | -0.0159 | No |
| 174 | MID1IP1 | na |  |  | 16790 | -1.024 | -0.0179 | No |
| 175 | STAU1 | na |  |  | 16818 | -1.032 | -0.0123 | No |
| 176 | KATNA1 | na |  |  | 16922 | -1.062 | -0.0105 | No |
| 177 | FGD6 | na |  |  | 17002 | -1.090 | -0.0072 | No |
| 178 | CDC27 | na |  |  | 17136 | -1.124 | -0.0066 | No |
| 179 | CAPZB | na |  |  | 17251 | -1.161 | -0.0047 | No |
| 180 | STK38L | na |  |  | 17314 | -1.189 | 0.0002 | No |
| 181 | NCK1 | na |  |  | 17360 | -1.202 | 0.0060 | No |
| 182 | ARF6 | na |  |  | 17460 | -1.240 | 0.0092 | No |
| 183 | KIFAP3 | na |  |  | 17601 | -1.294 | 0.0107 | No |
| 184 | NEDD9 | na |  |  | 17722 | -1.343 | 0.0135 | No |
| 185 | NET1 | na |  |  | 17938 | -1.460 | 0.0121 | No |
| 186 | UXT | na |  |  | 18439 | -1.790 | -0.0020 | No |
| 187 | YWHAE | na |  |  | 18585 | -1.908 | 0.0034 | No |
| 188 | SOS1 | na |  |  | 18592 | -1.915 | 0.0161 | No |
| 189 | ARHGAP5 | na |  |  | 18870 | -2.312 | 0.0173 | No |
Table: GSEA details [plain text format]

  

Fig 2: HALLMARK\_MITOTIC\_SPINDLE      
 Blue-Pink O' Gram in the Space of the Analyzed GeneSet

  

Fig 3: HALLMARK\_MITOTIC\_SPINDLE: Random ES distribution      
 Gene set null distribution of ES for **HALLMARK\_MITOTIC\_SPINDLE**
